# Was Jack the Ripper a Slaughterman? Human-Animal Violence and the World’s Most Infamous Serial Killer

**DOI:** 10.3390/ani7040030

**Published:** 2017-04-10

**Authors:** Andrew Knight, Katherine D. Watson

**Affiliations:** 1 Centre for Animal Welfare, Faculty of Humanities and Social Sciences, University of Winchester, Sparkford Road, Winchester SO22 4NR, UK; 2 School of History, Philosophy and Culture, Oxford Brookes University, Tonge Building, Gipsy Lane, Oxford OX3 0BP, UK; kwatson@brookes.ac.uk

**Keywords:** serial murder, Jack the Ripper, slaughterman, slaughterhouse, abattoir, slaughter, human–animal violence, history of crime, forensic medicine, animal welfare

## Abstract

**Simple Summary:**

The identity of Jack the Ripper remains one of the greatest unsolved crime mysteries in history. Jack was notorious both for the brutality of his murders and also for his habit of stealing organs from his victims. His speed and skill in doing so, in conditions of poor light and haste, fueled theories he was a surgeon. However, re-examination of a mortuary sketch from one of his victims has revealed several key aspects that strongly suggest he had no professional surgical training. Instead, the technique used was more consistent with that of a slaughterhouse worker. There were many small-scale slaughterhouses in East London in the 1880s, within which conditions were harsh for animals and workers alike. The brutalizing effects of such work only add to concerns highlighted by modern research that those who commit violence on animals are more likely to target people. Modern slaughterhouses are more humane in some ways but more desensitizing in others, and sociological research has indicated that communities with slaughterhouses are more likely to experience the most violent of crimes. The implications for modern animal slaughtering, and our social reliance on slaughterhouses, are explored.

**Abstract:**

Hundreds of theories exist concerning the identity of “Jack the Ripper”. His propensity for anatomical dissection with a knife—and in particular the rapid location and removal of specific organs—led some to speculate that he must have been surgically trained. However, re-examination of a mortuary sketch of one of his victims has revealed several aspects of incisional technique highly inconsistent with professional surgical training. Related discrepancies are also apparent in the language used within the only letter from Jack considered to be probably authentic. The techniques he used to dispatch his victims and retrieve their organs were, however, highly consistent with techniques used within the slaughterhouses of the day. East London in the 1880s had a large number of small-scale slaughterhouses, within which conditions for both animals and workers were exceedingly harsh. Modern sociological research has highlighted the clear links between the infliction of violence on animals and that inflicted on humans, as well as increased risks of violent crimes in communities surrounding slaughterhouses. Conditions within modern slaughterhouses are more humane in some ways but more desensitising in others. The implications for modern animal slaughtering, and our social reliance on slaughterhouses, are explored.

## 1. The World’s Most Infamous Serial Killer

In 1888, the streets of London were rough and poorly lit. In Whitechapel and surrounding areas, times were hard, and work started early for some. Others worked throughout the night in trades licit—such as the meat industry—and illicit. At around 03:40 on 30 August, a cart driver named Charles Cross was on his way to work when he spied what appeared to be a body lying in front of a gated stable entrance [1] (p. 27). A surgeon subsequently summoned estimated that part-time prostitute Mary Ann “Polly” Nichols had died only some 10 minutes earlier. Her throat had been cut twice and her abdomen mutilated with a series of violent, jagged incisions [1] (pp. 35, 47). Although unaware of it at the time, those present were witnessing what would subsequently be considered the first known work of the world’s most infamous serial killer—Jack the Ripper.

In the next 10 weeks, four other local women would share Polly’s fate. All were prostitutes who lived and worked in the impoverished slums of East London, and all but one had their throats cut prior to abdominal mutilation. As police efforts to identify the killer(s) remained unsuccessful, a climate of fear descended upon the city. A vigilante committee was formed, whose efforts were similarly fruitless. The murderer was never identified, with four subsequent brutal killings in Whitechapel—ending in 1891—being attributed to others. Jack’s final victim was considered to be Mary Jane Kelly, whose severely mutilated body was discovered on 9 November 1888 [2]. 

The extraordinary brutality of the murders, combined with the inability of the police or others to catch the perpetrator and consequent widespread media coverage, resulted in a fascination with the case that remains strong to this day. Hundreds of theories about Jack’s identity now exist [3], ranging from the credible to the bizarre. One recent theory asserts that “Jack” may have, in fact, been a woman. DNA swabs from letters sent to police at the time, some of them believed to have been from the actual killer, were not conclusive, but nevertheless led Ian Findlay, an Australian professor of molecular and forensic diagnostics, to assert the possibility that the Ripper might have been female [4]. However, the victims were all physically overpowered, and swiftly had their throats cut, in populated areas, whilst making barely a sound, suggesting that Jack possessed considerable physical strength.

Another theory suggests that Jack was in fact the estranged husband of the fifth and final victim, Mary Jane Kelly, and that the first four victims were used as decoys. By day, Francis Spurzheim Craig was a courtroom reporter, and a contemporary sketch of an inquest into the Ripper’s earlier murders is thought to show Craig sitting near the front of the court, reporting on a murder he himself might have committed. If true, it could be the only surviving image of the Ripper’s face. In a final twist, Craig committed suicide by slashing his own throat with a blade—in a very similar manner to the style in which Jack dispatched his victims [5]. Suicide has always been a possible explanation for the sudden cessation of Jack’s murders, along with other causes of death, unrelated imprisonment, institutionalization and emigration [6] (p. 371).

And yet, on several of his victims, Jack showed a particular propensity for anatomical dissection with a knife—and in particular, the rapid location and removal of specific organs. Only 14 minutes passed between successive patrols of Police Constable (PC) Watkins through Mitre Square after midnight on 30 September 1888. But within that short space of time, Jack was able to manoeuvre his fourth victim, Catherine Eddowes, into a darkened corner of the square, render her unconscious, cut her throat, pull up her clothing, and create a massive incision from groin to breastbone. Working in relative darkness, he then successfully located her uterus and left kidney and excised them. The difficulty of this task—even in good light, with a cadaver on a dissecting table at waist height—results from the location of the kidney, in particular. It lies buried within fat at the rear of the abdomen, beneath the stomach and other abdominal organs. And yet, in very poor light, and with the body awkwardly positioned at ground level, Jack nevertheless successfully completed this task with a speed that would have put most medical students to shame. He then created several relatively delicate cuts to her face, and had vanished into the night by the time PC Watkins reappeared. 

Indeed, with the exception of the third victim, Elizabeth Stride, whose attacker may have been interrupted, only the first victim Mary Ann Nichols was *not* bereft of any organs. The second victim, Annie Chapman, also had her uterus removed, and the final victim, Mary Jane Kelly, had her heart removed. Her body was also eviscerated, and her face severely mutilated.

Such obvious skills with the knife and, apparently, with anatomy, led commentators such as examining pathologist Phillips to conclude that “the murder could have been committed by a person who had been a hunter, a butcher, a slaughterman, as well as a student in surgery or a properly qualified surgeon” [6] (p. 246). Discomfiting though it is to one of us (AK) who is a member of the profession, he could even have been a veterinary surgeon. At least, in theory.

## 2. Not a Surgeon

**Figure 1:**
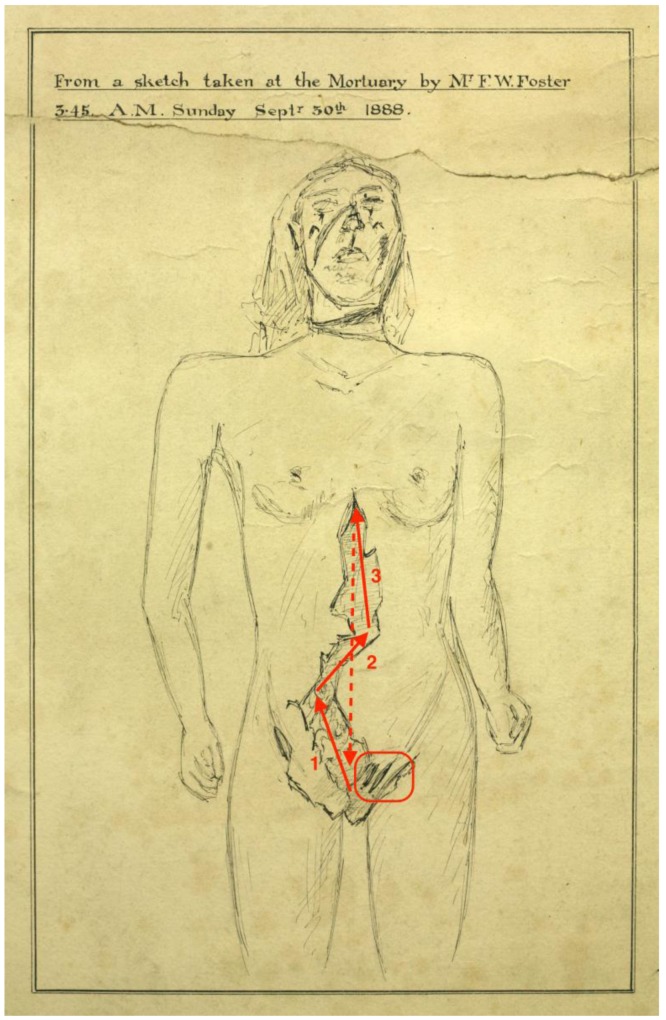
Analysis of wounds inflicted on Catherine Eddowes (From a sketch by Frederick William Foster, presented at the inquest held on 6 October 1888. Reproduced by permission of the Royal London Hospital Archives and Museum (Accession reference: RLHT1997/42). We have added locations of the exploratory incisions, along with the three primary incisions (solid) and the normal location and direction of the abdominal incision used for organ procurement (dashed) [7] (p. 21)).

However, examination of a mortuary sketch of the wounds inflicted on Catherine Eddowes’ body (Figure 1) provides several strong indications that Jack was unlikely to have been a medically-trained professional, or a medical or veterinary student.

It appears that Jack commenced by placing several exploratory incisions within Eddowes’ lower abdominal region. These were displaced from the midline, and hence incorrectly located for opening the abdomen for general organ procurement, which normally utilises an abdominal midline approach [7] (p. 21). 

**Figure 2:**
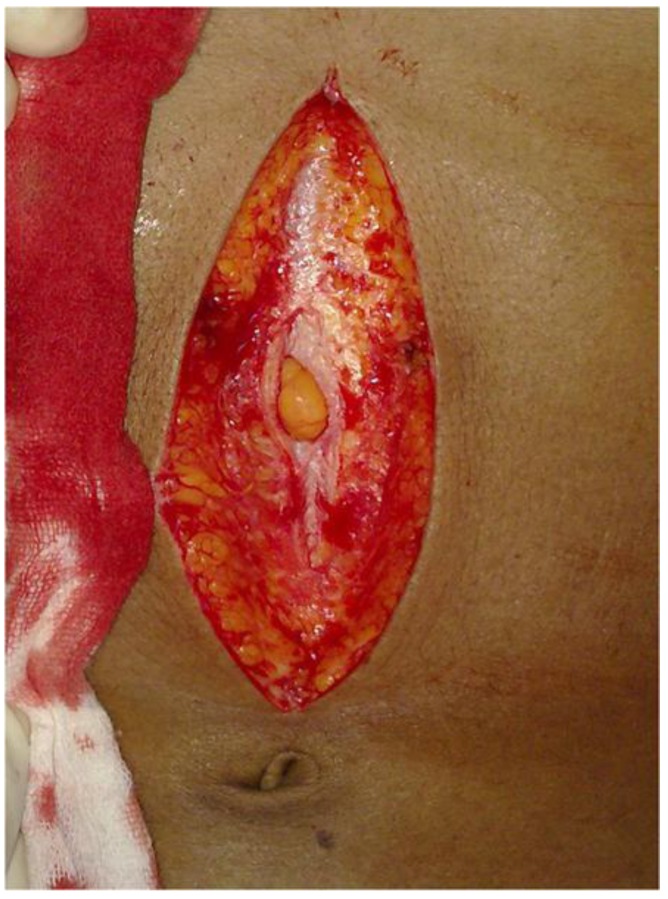
Normal abdominal surgical approach releasing skin tension following incision [8].

Of course, it is possible that these “exploratory” incisions may have been placed after the main incision (Figure 1—three solid lines). However, the careful placement of these exploratory incisions makes this extremely unlikely. They are very close to each other, and are parallel. To place these incisions in such careful proximity in darkness and haste, it would have been necessary for the skin to remain very stable. This stability would have been provided by the normal tension of the skin, which would have been released as soon as the first major incision was made (Figure 2). Thereafter, it would have been difficult to place those small incisions with the deliberateness of location apparent in the sketch of Eddowes’ body—and particularly in the case of one exploratory incision, directly next to the wound edge. Hence, it appears that these small incisions must have been placed prior to the main incision—i.e., they were indeed exploratory incisions which preceded the main incision.

Having established that these were exploratory incisions, their location indicates the most likely point of origin of the main incision. All were in the region of the groin. It is most probable that the main incision started as an additional exploratory incision in the same region, which was then extended in three major cuts toward the sternum (Figure 1—solid lines). 

However, when procuring organs, surgeons normally commence their abdominal incisions in the region of the sternum xiphoid process (i.e., the “head” end of the abdomen), and proceed toward the os pubis (i.e., the “feet” end) (Figure 1—dashed line) [7] (p. 21). Hence, it appears that the main incision to Eddowes’ abdomen proceeded in the direction opposite to that used by trained surgeons.

**Figure 3:**
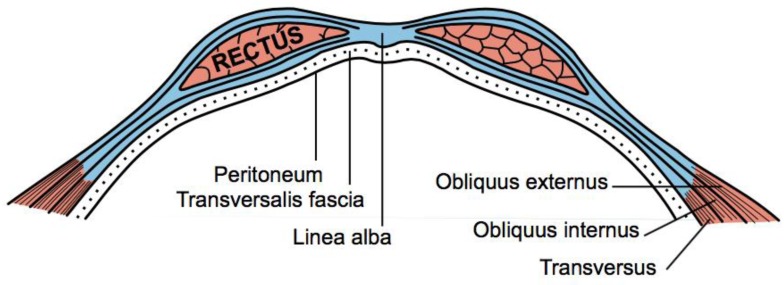
The linea alba is formed by the fusion of the aponeuroses surrounding the left and right rectus abdominis muscles [9].

**Figure 4:**
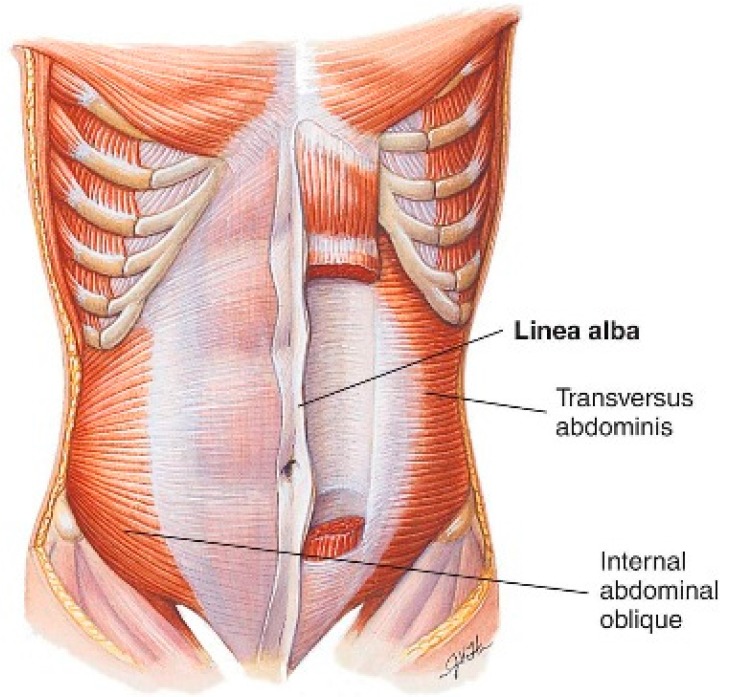
The linea alba runs the length of the abdominal midline from the xiphoid process to the pubic symphysis [10] (This article was published in Anatomy & Physiology, Thibodeau GA & Patton KT, p. 325, Copyright Elsevier (1996)).

Additionally, when performing a routine exploratory surgical approach to the abdomen, surgeons normally prefer the vertical midline approach, as it allows access via the *linea alba*—or “white line”—formed by the fusion of the aponeuroses (fibrous, fascial sheaths) surrounding the abdominal muscles—particularly the left and right rectus abdominis muscles (Figure 3 and Figure 4). 

An incision directly through this fascial line running vertically down the abdominal midline minimizes bleeding which would otherwise obscure the surgical field, and which could also potentially endanger the patient through blood loss. An incision in this location provides optimal exposure of the abdominal contents, and is preferred when abdominal exploration is desired. It also allows subsequent suture placement during wound closure within tough fibrous tissue, rather than within muscle. The latter is more fragile, and sutures are more likely to pull through under tension and the pressure applied by intra-abdominal contents, during the convalescent period [11] (p. 99). 

For these reasons, correct placement of the primary abdominal incision directly through the linea alba, without deviation to either side, is so important that surgeons go to considerable effort to achieve it. The importance of this fundamental principle is deeply instilled within surgical students during basic training, to the point that it becomes automatic. Surgical texts from the 1880s do not provide the details of basic surgical principles commonplace within modern texts, but those historical texts consulted give no reason to believe this fundamental surgical principle would not have been similarly taught to the surgery students of the day [12,13,14]. Indeed, any deviation from the linea alba during routine abdominal surgical exploration in the 1880s would have immediately resulted in the same bleeding and other adverse consequences that would occur today, providing immediate feedback to the surgeon and a strong compulsion to return to the linea alba.

Admittedly, it is normal that the loss of skin tension and parting of the skin edges following commencement of any incision makes it difficult to keep to a straight line. Nevertheless, the degree of raggedness of the main incision apparent on Eddowes is quite severe. It exceeds that which would be probable in anyone possessed of even minimal surgical skill, and even when working under conditions of poor light and haste. The incorrect placement of the exploratory incisions, incorrect direction of the primary incision, and the degree of deviation from the abdominal midline—not to mention the raggedness of the primary incision—strongly suggest that it was highly unlikely that Jack possessed even minimal surgical training or experience.

## 3. Anatomical Skill Required

The mutilations inflicted on Eddowes were, however, consistent with someone familiar with more general principles of opening an abdomen, and—based on the organs subsequently removed in darkness and haste—familiar with rapid location and identification of certain desired organs, which would be difficult or impossible for the inexperienced to locate amongst the liver, pancreas, stomach, bladder, small bowel, colon, uterus, ovaries, mesenteries, ligaments, vessels, nerves, ureters, urethra and intraabdominal fat [7] (p. 33). Phillips opined that the weapon was very sharp, and the way it was used seemed to indicate great anatomical knowledge. Hence, Jack may well have been, as Phillips noted, a “hunter, butcher, a slaughterman”, but most probably not “a student in surgery or a properly qualified surgeon” [15] (p. 109).

These sentiments were echoed by Frederick Gordon Brown (surgeon for the City Police) at the inquest on 5 October, 1888. Brown noted that certain organs were missing from the second and fourth victims, Annie Chapman and Catherine Eddowes, respectively. As reported in the *Denbighshire Free Press*, “Browne stated that the clever manner in which the left kidney and the other organ were removed betokened that the murderer was well versed in anatomy, but not necessarily in human anatomy, for he could have gained a certain amount of skill as a slaughterer of animals” [16].

However, the level of anatomical skill necessary for the excision of these organs was considered controversial by the experts of the day. At the autopsy of Catherine Eddowes, Phillips and Brown found that the injuries could have been inflicted by a person who had been a hunter, butcher, slaughterman, or a qualified or student surgeon [15] (p. 208). The mutilations were considered to have been inflicted by a sharp pointed knife, six inches in length. Some anatomical knowledge was considered necessary for removal of the left kidney, which can easily be overlooked. Such knowledge might have been possessed by someone in the habit of cutting up animals [15] (p. 229). However, the first doctor on the scene, George William Sequeira, did not think the perpetrator possessed any great anatomical skill, or that he had designs on any particular organ. City Public Analyst William Sedgwick Saunders was present at the autopsy and agreed with both of these points [15] (p. 232).

As reported in the *South Wales Echo* (27 September 1888), the coroner observed with respect to Annie Chapman, that, “… the uterus has been taken away. The body has not been dissected, but the injuries have been made by someone who had considerable anatomical knowledge and skill. There are no meaningless cuts. The organ has been taken away by one who knew where to find it, what difficulties he would have to contend against, and how he should use his knife so as to abstract the organ without injury to it. No unskilled person could have known where to find it, or have recognised it when it was found. For instance, no mere slaughterer of animals could have carried out these operations. It must have been someone accustomed to the post mortem room” [17].

As the coroner noted, a “mere slaughterer” might not have possessed the necessary dissection skills and anatomical knowledge. However, an abattoir worker experienced in both animal slaughter and butchery might well have done.

## 4. Could Jack Have Been a Slaughterman?

An intriguing array of other factors lend credence to the theory that Jack might have been a slaughterman. First, in 1888, there were many slaughterhouses in the Whitechapel area. Their presence stemmed from the appetite of the sizeable and growing London populace for meat, and from the lack of the modern refrigeration, transportation infrastructure and large-scale mechanization that characterize modern slaughtering and allow it to be conducted far removed from urban areas [18].

Indeed, the neighbouring borough of Islington was the centre of the live meat market in London at the time. As many as 10,000 cattle were on sale daily, and there was an account of 38,500 sheep being gathered for a single market. As reported in the *Birmingham Daily Post* in 1888, “In 1887, there were sold at Islington, for London consumption, 235,762 cattle, 809,914 sheep, 13,349 calves, and 1119 pigs”. And yet, the 1887 market was recovering from a depression. In 1864, the even greater figure of 346,000 cattle were sold [19]. 

These enormous numbers of animals being consumed by the growing London population required a similarly sizeable slaughtering capacity. Victorian slaughterhouses were not the vast, industrialised killing and butchering factories of today, and workers were not assisted by machines. Instead, there were a very large number of small facilities.

Indeed, the body of Jack’s first victim, Mary Ann Nichols, was discovered by a police constable (John Neil) in Buck’s Row, very close to one of these establishments. As he reported in *The Flintshire Observer*, “The first to arrive on the scene after I had discovered the body were two men who work at a slaughterhouse opposite” [20]. Another then joined them. These men were reported as Henry Tomkin, horse slaughterer, and two companions, James Mumford and Charles Brittain, who worked for Messrs. Barker & Co. (Whitechapel, London, UK), Horse Slaughterers, in Winthrop Street, adjoining Buck’s Row and only 150 yards from Nichols’ body [21] (p. 102). 

As noted by Odell, “Well practiced in the art of cutting throats, legitimately owning and carrying knives, working and probably living in or near Whitechapel and Spitalfields, the Ripper as a slaughterman made a great deal of sense. Moreover, he would have blended perfectly with the local colour and character of the streets…” [21] (pp. 102–103). This point is affirmed by Samuel Barnett in a letter published in *The Times* (19 September 1888): “At present animals are daily slaughtered in the midst of Whitechapel, the butchers with their blood stains are familiar among the street passengers, and sights are common which tend to brutalize ignorant natures” [22]. Working hours and conditions in 1888 were far removed from those of today, and animals were frequently slaughtered at night—facilitating the nocturnal presence—and indeed alibis—of any slaughtermen in the area.

Odell noted that such an assailant would have frequented “pubs and lodging houses and (was) probably known to the prostitutes. He would have many attributes that made him an accepted and unexceptional character, beyond suspicion and creating no fear” [21] (p. 103). The latter point in particular would have assisted a perpetrator to have approached these women without challenge, possibly entice them into quiet locations, and successfully overpower them, before any had the chance to raise an alarm. Most of these murders occurred nearby to other people, yet never a sound was heard.

In 1966, some sketches relating to the murder of Catherine Eddowes were discovered in a basement of the London Hospital. Professor Francis Camps, an eminent pathologist with an interest in the case, published the sketches with his analysis in *The London Hospital Gazette.* Commenting on the fact that the victims evidenced facial congestion, died without calling out, and with possibly less blood loss than might have been expected, he suggested that strangulation—“a common practice of sexual murderers”—might have been the real cause of death [23] (p. 27). However, this was contested by the doctors of the time. For Elizabeth Stride, the cause of death was given as a severed left carotid artery and division of the trachea (windpipe); for Catherine Eddowes, haemorrhage from the left carotid artery; and for Annie Chapman, also haemorrhage [21] (pp. 109–110).

Regardless of the degree to which strangulation was used, considerable physical strength would have been necessary, to so quietly and quickly overpower Jack’s victims. Such strength would have been necessary for, and induced by, the very physical nature of the work in the slaughterhouses of this era. As described in the *Birmingham Daily Post*, “…a slaughterhouse is at its best but a chamber of horrors…in which oxen and sheep become beef and mutton under the hands of the brawny, half-naked pole-axing men” [19]. Half-naked, no doubt, because of the hard physical labour involved, and the exercise-induced body temperature rises and sweating. 

Odell continues thus: “Added to this would be his knowledge of local geography… It does not take much imagination to visualize a slaughterman killing his human prey and escaping to the blood-soaked sanctuary of the abattoir where he worked. Indeed, what better place could there be to hide the murderer and to conceal his murder weapon and visceral trophies” [21] (p. 103)?

The suspicion that Jack was intimately familiar with the local geography of the area was reinforced by a description of his likely movements, recited by Professor Camps in *The London Hospital Gazette* (1966). After murdering Catherine Eddowes in Mitre Square, Camps noted that Jack must have travelled across Houndsditch and Middlesex Street to Goulston Street where in a passage leading to a staircase of some flats, a blood-stained piece of Eddowes’ apron was found by PC Alfred Long of H Division, Metropolitan Police, at 02:55, along with a message scrawled on a nearby doorway in chalk. Camps asserts that Jack would have then travelled north to Dorset Street, pausing there to wash blood off his hands at a public sink set back from the street. Camps asserted that Jack’s knowledge of the sink location in particular suggested that he knew the neighbourhood [23]. 

## 5. Might Jack Have Been a Shochet?

Odell continued that, “If throat-cutting was Jack the Ripper’s hallmark, there was one particular kind of slaughterman whose calling required perfection in this special skill. That was the Jewish ritual slaughterman, or shochet” [21] (p. 103).

And in fact, there was a significant need for Jewish ritual slaughter in nineteenth-century London. As Odell noted, there was a large Jewish population—estimated at 60,000—in the East End of London in 1889. Hence, there would have been a significant number of abattoirs conducting Jewish ritual slaughter (shechita), and a significant number of highly experienced Jewish ritual slaughtermen (shochetim) [21] (pp. 103, 105).

Shechita is the only method of producing meat and poultry allowed by Jewish law. Such meat is then designated as “kosher”, signifying that it complies with the regulations of kashrut (Jewish dietary law). As described by Shechita UK, “Shechita is performed by a highly trained shochet. The procedure consists of a rapid and expert transverse incision with an instrument of surgical sharpness (a chalaf), which severs the major structures and vessels at the neck… The frontal structures including the trachea, oesophagus, the carotid arteries and jugular veins are severed in a rapid and uninterrupted action” [24] (pp. 3, 5). Velarde et al. provided further details: “During religious slaughter, animals are killed…by a transverse incision across the neck that is cutting the skin, muscles (brachiocephalic, sternocephalic, sternohyoid, and sternothyroid), trachea, esophagus, carotid arteries, jugular veins and the major, superficial and deep nerves of the cervical plexus” [25] (p. 278).

Such an incision is highly consistent with the neck incisions found on Jack’s victims. Henry Llewellyn, the doctor called to examine the body of Mary Ann Nichols at the scene of her death, conducted a post-mortem examination later that morning. He reported that there was an incision about eight inches long running from a point below the left ear, rostrally (frontwards) around the neck, to a point below the right jaw, which had completely severed most of the tissues, including the large vessels (jugular veins and carotid arteries) on both sides of the neck. As he reported in *The Flintshire Observer*, “These cuts must have been caused with a long-bladed knife, moderately sharp, and used with great violence” [20].

Very similarly, when testifying at the inquest into the death of Catherine Eddowes, Dr Brown described her throat incision, thus: “The throat was cut across to the extent of about 6 or 7 inches. A superficial cut commenced about an inch and ½ below the lobe about 2½ inches behind the left ear and extended across the throat to about 3 inches below the lobe of the right ear. The big muscle across the throat was divided through on the left side—the large vessels on the left side of the neck were severed—the larynx was severed below the vocal chords (*sic*). All the deep structures were severed to the bone the knife marking intervertebral cartilages—the sheath of the vessels on the right side was just opened” [21] (p. 105). 

Additionally, as reported by Shechita UK, the shochet’s duties do not end there: “He examines the organs and vessels immediately after severance by the shechita incision, to ascertain that the shechita was properly performed, this examination is visual and tactile (b’dikath ha’simanim). This integral part of the shechita process is required by Halacha (Y.D. 25:1). The shochet also examines the internal organs and lungs (b’dikath ha’reyah) of an animal in order to ascertain whether there are any abnormalities or defects disqualifying the animal from being kosher (Y.D. 29-60)” [24] (p. 7). (Y.D. refers to the Yoreh Deah, or Codes of Jewish Law) (As reported by Shechita UK, “Jewish laws governing shechita and the animal welfare considerations are to be found in the Talmud (Oral Law of Judaism) Tractate Chullin, Mishneh Torah of Maimonides, the Shulchan Oruch: Yoreh Deah (Codes of Jewish Law) by Rabbi Joseph Karo …” Additionally, Halacha refers to Jewish law and jurisprudence, based on the Talmud [24] (p. 6)).

Hence, any experienced shochet is likely to be highly skilled at the type of neck incisions found on Jack’s victims. They are also likely to be highly skilled at visual and tactile examination of internal organs. This familiarity—and in particular, tactile familiarity—would have been extremely helpful to an assailant seeking to rapidly locate and remove specific organs from the bodies of his victims, in poor lighting.

The names and details of licenced shochetim were held by the London Board of Shechita. Unfortunately, however, their records for the relevant time period were destroyed by the German bombing of London in 1940 [21] (p. 105). 

But of course Jack was not necessarily a shochet. As Odell neatly surmised, “an ordinary slaughterman possessed all the skills demonstrated by the Ripper’s knife work”, although, “the Shochet had the edge when it came to throat-cutting” [21] (p. 106).

## 6. Analysing Jack’s Words

**Figure 5:**
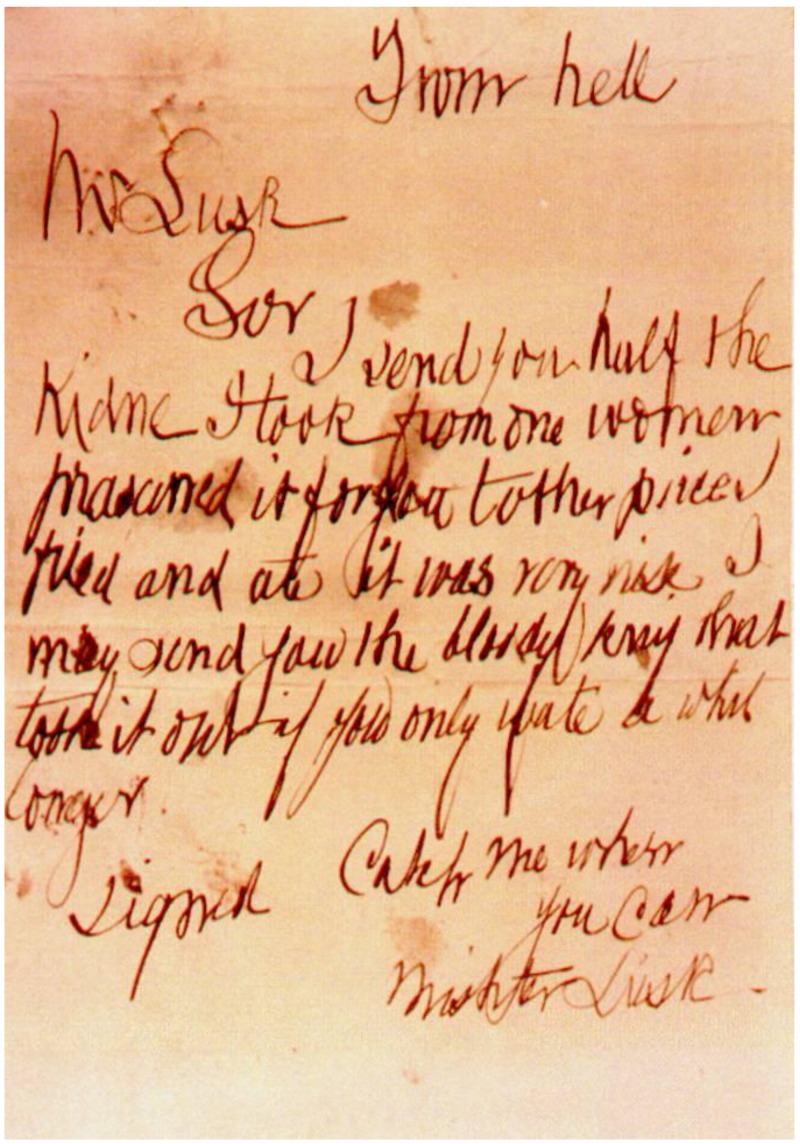
Letter purportedly from Jack the Ripper (Anon. (n.d.). A photographic copy of the now lost “From Hell” letter, postmarked 15 October 1888 [27]).

Jack’s crimes were the centre of public attention in nineteenth-century London, and many letters were received by the authorities purporting to be from the Ripper. Most are believed to have been hoaxes. However, a specimen accompanying one such letter (Figure 5) suggests authenticity. 

On 16 October, 1888, Mr George Lusk, Chairman of the Whitechapel Vigilance Committee, received by post a cardboard box containing a portion of a human kidney preserved in ethanol (“spirits”). Openshaw, Pathological Curator of the London Hospital, subsequently examined it. He reportedly stated that it belonged to a woman of about 45 (Eddowes was 46), and had been removed within the preceding three weeks (Eddowes was killed a fortnight prior). This specimen reportedly matched Eddowes’ missing left kidney, because two inches of renal artery remained in the victim’s body, whilst the third inch was attached to the kidney, and because both the right kidney remaining in the body, and the left kidney received by post, showed signs of severe Bright’s disease [23] (pp. 33–34). However, as with many historical details pertaining to the Ripper’s murders, some of these details are contested. For example, police surgeon Dr Brown reportedly stated that the kidney had been trimmed up, and that the renal artery was entirely absent [1] (p. 189), [26] (p. 168). 

Nevertheless, given the timing of the letter, and its accompanying specimen, this is the letter most likely to have been an authentic communication from Jack himself. Entitled “From hell”, the letter stated,

“Mr Lusk
Sor
I send you half the
Kidne I took from one women
prasarved it for you tother piece I
fried and ate it was very nise. I
may send you the bloody knif that
took it out if you only wate a whil
longer
Signed Catch me when
you can
Mishter Lusk”

Several other communications received were addressed to “Dear Boss”, or refer to the reader as “Boss” [23] (p. 33). However, none were similarly accompanied by crime scene artefacts, and it is unknown which, if any, were authentic. 

The appalling standard of spelling and grammar within this letter suggest either that the author was neither a medical student nor professional, or that they possessed a rare degree of imagination. The misspelling of ‘kidney’ in particular is highly suggestive of the former. It may be difficult for non-medically trained readers to appreciate just how deeply the correct spelling of such words is ingrained in anyone with significant medical training, but having (in the case of one of us) been through such training, we can testify that this is indeed the case. It is far more likely the letter was authored by a person who lacked the benefit of more than the most elementary degree of education. Similarly, “Boss” is an honorific commonly used by people in jobs with low socioeconomic status—although, as stated, it is unknown whether any of the letters addressed to “Boss” were authentic.

## 7. Socioeconomic Factors

Living conditions in East London were relatively impoverished at the time, and as noted previously, the livestock market was recovering from a depression. It was far from a desirable locale in which to live and work. As noted by Superintendent Thomas Arnold: “…a considerable portion of the population of Whitechapel is composed of the low and dangerous classes, who frequently indulge in rowdyism and street offences” [15] (p. 314).

This was reinforced by the sentiments of Mr Samuel Barnett, Vicar of St Jude’s, as paraphrased by James Monro, Metropolitan Police Commissioner, in Evans and Skinner: “Vice of a very low type exists in Whitechapel—such vice manifests itself in brawling and acts of violence which shock the feelings of respectable persons...The facility with which the Whitechapel murderer obtains victims has brought this prominently to notice, but to anyone who will take a walk late at night in the districts where the recent atrocities have been committed, the only wonder is that his operations have been so restricted. There is no lack of victims ready to his hand, for scores of these unfortunate women may be seen any night muddled with drink in the streets and alleys, perfectly reckless as to their safety, and only anxious to meet with anyone who will help them in plying their miserable trade” [15] (p. 495).

Barnett further reported in a letter to *The Times* (1889), that, “…the streets still offer almost every night scenes of brutality and degradation. A body of inhabitants…have patrolled the neighbourhood during the last nine months on many nights every week between the hours of 11 p.m. and 3 a.m. Their record tells of rows in which stabbing is common, but on which the police are able to get no charges; of fights between woman stripped to the waist, of which boys and children are spectators; of the protection afforded to thieves, and of such things as could only occur where opinion favours vice” [28].

However, it is not only humans that may fall victim to poor socioeconomic circumstances and any related tendencies toward violent behaviour. Animals may also be affected. In 2010, veterinarian Peter Wedderburn noted that times of economic hardship appear to result in greater numbers of companion animals being abandoned and mistreated, including within organized fights, and by drowning, stabbing, burning or neglect [29].

Prior to the implementation of modern worker protection laws and policies, and bereft of the benefits of modern machinery, slaughterhouse work would have been even harder and more dangerous than it is today, and the pay—particularly in the depressed boroughs of nineteenth-century East London—would have been low. Meat, milk and eggs were relatively more expensive than they have become today, with the benefits of modern industrialisation, mass production and market pressures [30].

Within the socioeconomically depressed environment of East London in the 1880s, it is easy to imagine workers appropriating small quantities of meats as they worked, to be taken home for later cooking and consumption. To a worker in the habit of regularly committing such petty thefts, taking home a kidney for cooking and consumption would have represented a far smaller step, that sprang to mind far more naturally, than might have occurred to a murderer from some other background.

## 8. Slaughterhouse Impacts on Workers

Even modern-day slaughterhouses are relatively dangerous places to work, with unusually high rates of injury and poor health. As Jacques (2015) put it, “The reality of slaughterhouse work is that this multi-billion dollar industry employs thousands of people who work for low wages in physically dangerous jobs” [31].

As reported by Cohidon et al. (2009) in a study of 3000 French meat industry employees: “Their risk of accidents is high, especially in slaughtering and cutting large animals; this is among the most dangerous of all French occupations. The use of knives and dangerous machines, the movements and postures required, and slips and falls cause most accidents” [32] (p. 808). They noted that “Other countries have made similar observations in the past several years”. In a recent study of Estonian slaughterhouse workers, Kristina Mering confirmed this: “The workers are subject to rapid repetitive movements, incurring blisters and stiffness, having to work in heat and cold with really sharp knives which can cause accidents. All agreed that they were underpaid for the work that they do” [33].

Cohidon and colleagues also demonstrated poor perceived health among the meat industry employees studied. Working hours disrupting sleeping rhythms were perceived to be a contributing factor [32]. This would certainly have been a factor in the meat industry of East London in the 1880s, and indeed, Jack’s murders all occurred during the small hours of the night.

## 9. Slaughterhouses and Violent Crime

Although conditions in modern slaughterhouses are usually better for both workers and animals, they are hardly conducive to the physical or psychological health of either. Unfortunately, it is not only the workers themselves that are affected. After analysing 1994–2002 data from 581 U.S. counties, Fitzgerald et al. (2009) reported that total arrest rates, and arrests for violent crimes, rape and other sex offenses were increased among slaughterhouse workers, when compared to those from other industries [34]. 

Comparative data were sourced for industries that were similar in the senses that they also had high immigrant worker concentrations, were also manufacturing industries (with the exception of one, which was included due to a high rate of immigrant concentration), and were similarly characterized by low pay, routinized labour, and dangerous conditions [35,36,37,38]. These were the iron and steel forging, truck trailer manufacturing, motor vehicle metal stamping, sign manufacturing, and industrial laundering industries. 

After controlling for the number of young men in the county, population density, the total number of males, the number of people in poverty, international migration, internal migration, total non-White and/or Hispanic population, the unemployment rate, and the total county population, the authors found that slaughterhouse employment had significant positive (and “unique”) effects on the arrest and report rate scales, as well as on rates of total arrests, arrests for violent crimes, arrests for rape, and arrests for other sex offences. In fact, the expected arrest and report values in counties with 7500 slaughterhouse employees were more than double the values where there were no such employees. 

Of the comparison industries, only one (truck trailer manufacturing) had a significant effect on the total arrests variable, but it was a negative effect. Two of the comparison industries (truck trailer manufacturing and motor vehicle metal stamping) had significant effects on violent arrests, but both were negative effects. Similarly, only one comparison industry (iron and steel forging) demonstrated a significant effect on arrests for rape, but once again, it was a negative effect [34]. 

The authors noted that, although “employment in the manufacturing sector in general has suppressant effects on crime (e.g., Lee and Ousey, 2001); this is clearly not the case for the slaughterhouse subsector of manufacturing” [34] (p. 175).

Similarly, Jacques analysed 2000 data from 248 U.S. counties in states chosen for their concentrations of cattle slaughterhouses and employment within them. After controlling for key variables in the social disorganization literature, she found that slaughterhouse presence in the counties studied corresponded with a 22% increase in total arrests, a 90% increase in offenses against the family, increased aggravated assaults, and a 166% increase in arrests for rape [31].

Particularly noteworthy was the significant positive effect of slaughterhouse employment on sexual assault rates, revealed by Fitzgerald et al. (2009). Offences within this category included sexual attacks on males, incest, indecent exposure, statutory rape, and “crimes against nature”. As these authors revealed, “Increases in slaughterhouse employment had a significant positive effect on rape arrests across the entire time period under study” [34] (p. 174).

## 10. Human-Animal Violence Links

Fitzgerald et al. (2009) observed that, “Many of these offenses are perpetrated against those with less power, and we interpret this as evidence that the work done within slaughterhouses might spillover (*sic*) to violence against other less powerful groups, such as women and children” [34] (p. 174). Indeed, most of the increases in violent crime rates surrounding slaughterhouses have been attributed to increases in domestic violence and child abuse [39,40] (p. 40), [41] (p. 103). 

Links between the commission of violence toward vulnerable groups, including women, children and the elderly, and the commission of violence toward animals, have been extensively documented since the first case report published in 1806, describing an adult male who was violent toward both animals and humans [42]. 

One of the first researchers in this area was MacDonald, who in 1961 became the first to explore the links between childhood animal cruelty and later violence toward humans. By sampling 48 psychotic patients and 52 nonpsychotic patients, he identified three characteristics consistently found among the most sadistic individuals: enuresis, fire setting, and childhood cruelty toward animals. He subsequently concluded that this “triad” of behaviours could be a strong predictor of homicidal behaviour [43]. 

Three years later, Mead similarly noted that childhood animal cruelty could indicate the formation of a spontaneous, assaultive character disorder. Animal cruelty “could prove a diagnostic sign” and “… such children, diagnosed early, could be helped instead of being allowed to embark on a long career of episodic violence and murder” [44] (p. 22). 

Since the late 1970s, behavioural specialists at the Federal Bureau of Investigation’s Behavioural Science Unit have suspected that animal cruelty may be involved in the development of serial killers [45], and in 1988, an FBI study implicated animal cruelty as a possible early warning sign of serial murder [46]. 

In 1987, the American Psychiatric Association (APA) continued in this vein, adding animal cruelty as a symptom of childhood conduct disorders to the third (revised) edition of its *Diagnostic and Statistical Manual of Mental Disorders* [47]. The *DSM-IV* defined conduct disorder as “a repetitive and persistent pattern of behaviour in which the basic rights of others or major age-appropriate societal norms or rules are violated” [48] (p. 90). The APA suggested that many of these same children would progress to show similar symptoms during adulthood, and that some of these would meet the criteria for anti-social personality disorder. This has been continued within recent editions *DSM-IV* [49], and *DSM-5* [50]. 

A series of studies examining the relationship between childhood animal cruelty and later violence against humans have occurred in the last two decades. A number demonstrated such an association [45,51,52,53,54,55,56,57]. Ressler et al. (1998), for example, studied 36 male sexual murderers, 29 of whom were serial murderers. Of the 28 subjects for whom childhood background data were available, 36% had committed animal cruelty as children, 46% had committed animal cruelty as adolescents, and 36% had committed animal cruelty as adults. The authors concluded not only that cruelty to animals might predispose toward violence against humans later in life, but that it might also predict the most extreme forms of violence [55]. 

Merz-Perez et al. (2001) interviewed 45 violent and 45 nonviolent offenders incarcerated in a Florida maximum-security prison. Violent offenders were significantly more likely than nonviolent offenders (56% vs. 20%) to have committed acts of animal cruelty as children. Particularly significant, given the way in which Jack killed his victims, and the similarity to Jewish ritual slaughter, they discovered that the way in which violent offenders abused animals resembled the methods they subsequently used to commit crimes against their human victims [53]. 

Wright and Hensley (2003) investigated 354 cases of serial murder, finding that 21% of these murderers had engaged in animal cruelty. They also described, in chilling detail, five cases in which children suffering humiliation or abuse vented their repressed frustration and aggression on animals, and later “graduated” to become the well-known serial killers Carroll Cole, Jeffrey Dahmer, Edmund Kemper, Henry Lee Lucas, and Arthur Shawcross [45].

These cases were consistent with the so-called ‘violence graduation hypothesis’, which holds that early animal cruelty provides the individual with the opportunity to learn first-hand about violence, practice violence on available targets (animals), and be desensitized to the consequences of violent behaviour [58]. 

A number of studies have specifically focused on the commission of violence towards women, usually by surveying female victims of intimate partner violence during their visits to secure shelters. Overall, around 50% of women in violent relationships reported that their partner had hurt or killed one of their pets (e.g., 46% in Faver and Strand, 2003; 52.9% in Volant et al., 2008; 53% in Carlisle-Frank et al., 2004; 54% in Ascione et al., 2007; 57% in Ascione, 1998) [59,60,61,62,63]. Somewhat divergently, Flynn (2000) reported a comparatively lower figure of 26% [64]. 

Furthermore, batterers who also abuse pets appear to differ from those who do not in other ways as well. One study found that batterers who abused pets were more dangerous, and used more controlling behaviours, than men who had not abused pets [65]. Batterers who had committed pet abuse also exhibited higher rates of sexual violence, marital rape, emotional violence, and stalking.

On the other hand, one-fifth of children are believed to have participated in some form of animal cruelty [66], and the vast majority do not “graduate” to become serial killers. Unsurprisingly, therefore, other studies have shown no relationship between childhood animal cruelty and later violence against humans, or have refuted the notion that one comes before the other, i.e., a time order relationship [67,68]. 

In 1987, Felthous and Kellert published a meta-analysis of 15 studies from the 1970s and 1980s to examine the link between childhood animal cruelty and later violence toward humans. They found that most studies failed to indicate a relationship between childhood animal cruelty and later violence toward humans, and they described data which supported this association as being “soft and of dubious reliability” [69] (p. 69). 

However, nine of the ten studies that failed to find a clear link between childhood animal cruelty and later violence against humans analysed single acts of violence directed toward humans rather than recurrent violence—which limits their applicability to serial killers such as Jack the Ripper. In their survey of 180 inmates from one medium-security and one maximum security prison in 2007, Hensley et al. (2009) found that repeated acts of animal abuse in earlier life were significantly related to later repeated acts of interpersonal violence as an adult [52]. 

Other limitations of these 10 studies included lack of clear definitions of the behaviours being defined, and reliance on case records, rather than face to face interviews. Accordingly, significant concerns persist about the possibility that the commission of violence towards animals may predispose to similar behaviour towards humans.

As Flynn (2011) put it, “animal abuse and interpersonal violence do often go together. Animal abuse can be a risk factor, a marker, and sometimes a precursor of other forms of violence, and vice versa. … it is still important for judges, juries, prosecutors, clinicians, child protective workers, shelter workers, veterinarians, police, and legislators to take animal abuse seriously” [66] (p. 461). 

## 11. Slaughterhouse Work: Predisposing Toward Violence

There appears to be something about slaughterhouse work in particular, that predisposes toward violent crime. This was observed and documented as early as the turn of the twentieth century in his book *The Jungle* by Upton Sinclair, as recounted by Fitzgerald et al. (2009): “(Sinclair) exposed the devastating work conditions and living environments of those who toiled in Chicago’s stockyard slaughterhouses. In *The Jungle*, he made a connection between the numerous after-work fights instigated by slaughterhouse workers and the killing and dismembering of animals all day at work: “He (the police officer) has to be prompt—for these two-o’clock-in-the-morning fights, if they once get out of hand, are like a forest fire, and may mean the whole reserves at the station. The thing to do is to crack every fighting head that you can see, before there are so many fighting heads that you cannot crack any of them. There is but scant account kept of cracked heads in back of the (stock) yards, *for men who have to crack the heads of animals all day seem to get into the habit, and to practice on their friends, and even on their families, between times* (Sinclair, 1905/1946, pp. 18–19 emphasis added)”” [34] (p. 158). 

The other manufacturing industries studied by Fitzgerald et al. were similar in labour force composition, injury and illness rates, but different in one crucial way: the materials of production are inanimate objects, rather than living animals. As the authors noted, “…Rémy (2003) and Smith (2002) have demonstrated that the slaughterhouse occupies a contradictory position within society. Formal rules about requiring humane slaughter acknowledge that sentient creatures are being killed. Yet those who are engaged in the work of the slaughterhouse also develop constructions that allow them to carry out this work” [34] (p. 159). As Jacques (2015) put it, “The setting of slaughterhouse work promotes a disconnection between humans and nonhuman animals, one in which nonhuman animals are treated as “products” and the act of slaughtering a nonhuman animal is compartmentalized into separate tasks from the kill floor to the fabrication room” [31] (p. 3).

Animals killed in slaughterhouses are objectified to some degree as ‘things’ that may be killed, dismembered, repackaged and otherwise used for human purposes. Such constructions would have been even more easily acquired prior to the development of modern legislation requiring more humane slaughter, and that accordingly encouraged specific consideration of the need for humane treatment of animals.

The objectification of animals in these environments facilitates the suppression of the sympathy for animals, and concern for their interests, that would otherwise manifest more strongly. Such sympathy and concern would create greater psychological stress for slaughterhouse workers who are required to treat animals in ways very contrary to that morally compelled by their true status as sentient beings who have done us no wrong, and as creatures who are commonly fearful and stressed in these environments, and who do not wish to die.

In her recent study of Estonian slaughterhouse workers, Kristina Mering confirmed this suppression of natural sympathy: “In order for them to be able to do their work, they need to block out all emotions. Since they understand that they are taking the life of an animal, they need a strong blocking mechanism to keep thoughts like this out. They build a routine that numbs the emotions and lets them do their work without thinking about the killing. When I asked one person about the stabbing, they put it like this: “If we would think about it, it would be the wrong place to work”. They wear earphones and listen to music or the radio” [33].

Sociologist Erika Cudworth also confirmed this during her interviews with local authority inspectors and slaughterhouse workers in London from 1994 to 1995. She reported that, “According to those who teach the skill at Smithfield market (*sic*), the largest meat market in London, it takes a “certain kind of person” to slaughter, one who has “disregard for the lives of animals” and has “got to be callous” (interview, butcher and tutor, Smithfield Meat Market, November 1993)” [70] (p. 13).

If slaughterhouse workers allowed themselves to feel appropriate sympathy and concern for the animals, given their status as sentient beings who do experience fear, pain, stress and other forms of suffering, it could interfere with their abilities to fulfil the roles required of them, and hence, their economic survival. Given the low socioeconomic status of most slaughterhouse workers, and the limited employment prospects in many of the rural locations where abattoirs are located, this is no small matter today. However, in the depressed environment of nineteenth-century East London, where social security was almost non-existent, it could have significantly impacted chances of literal survival. Hence, the workplace pressures in these nineteenth-century slaughterhouses likely to result in objectification of, and decreased empathy for, the animals that were killed in them, were most probably even stronger than they are today.

The suppression of natural sympathy that might otherwise manifest affects both animals and workers. As noted by Dillard (2008), “By habitually violating one’s natural preference against killing, the worker very likely is adversely psychologically impacted” [71] (p. 401).

The rapid pace of many modern slaughterhouse production lines also places considerable pressure on workers, increasing rates of worker injury [72]. Pachirat (2011) exemplified the pressures in a description of the work in the liver-packer room of a beef slaughterhouse: “At the rate of one cow, steer, or heifer slaughtered every twelve seconds per nine-hour working day, the reality that the work of the slaughterhouse centers around killing evaporates into a routinized, almost hallucinatory blur. By the end of the day, by liver number 2394 or foot number 9576, it hardly matters what is being cut, shorn, sliced, shredded, hung, or washed: all that matters is that the day is once again, finally coming to a close” [73] (p. 138). 

It is understandable (if not excusable), that the rapid pace of slaughtering and processing required by high volume production processes, combined with the necessity of a callous mind-set toward the animals, and the minimal training, difficult conditions and low pay rates provided to workers, create conditions in which workers might vent their frustrations on the animals—when they baulk at strange or disturbing sounds, smells, environments, or at rapid, forceful handling, which cause delays and require physically tired workers to exert extra effort. Burt (2006) asserted that increasingly detached forms of the dispatching of animals are prompted more by concern for speed and thus profit, than for welfare [74]. In her study of London slaughterhouse workers, Cudworth (2015) reported verbal abuse of animals, and the use of electric goads used to hurry them. She stated that “violence towards domesticated animals is routinized, systemic and legitimated” [70] (p. 14).

Given such circumstances, it is perhaps unsurprising that Gail Eisenitz’s (1997) extensive study of U.S. slaughterhouses revealed alarming tales of systematic and normative cruelty against animals [75]. And indeed, despite commitments to humane practices and applicable legislation, abuses of animals within slaughterhouses remain prevalent even today. 

## 12. Implications for Modern Slaughterhouses

Socioeconomic conditions in East London in the 1880s were dire, and the harsh realities of the time were reflected in the slaughterhouses that were so numerous within its crowded and squalid streets [18]. As noted previously, Barnett (1888, p. 3) described the resultant deplorable scenes in *The Times*, concluding that “For the sake of both health and morals the slaughtering should be done outside the town” [22].

More than 125 years later, Barnett’s wishes have come true. Slaughtering is no longer conducted within urban centres, and has generally been removed to rural locations far less visible. The numerous small operations of the 1880s have been amalgamated into larger corporate enterprises. However, these have become vaster, more impersonal, industrialised systems of killing, within which animal abuse continues. 

Modern standards for humane slaughter do exist [76,77,78]. They acknowledge that after traveling what may be considerable distances to these much larger, modern, slaughterhouses, animals often arrive hungry, thirsty and exhausted. Significant social stress may result from mixing with unfamiliar animals. They prescribe that when lairaged in pens at the slaughterhouse, animals should be watered, fed, and protected from environmental extremes. Those that arrive ill should be treated or slaughtered without delay. Too often, however, this does not occur [79]. 

The races and approaches to the killing area should be designed to minimise fear and distress, animals should always be handled humanely, and the killing process should be conducted using humane restraint and effective stunning. Different species are killed using a variety of methods determined partly by their physical size and the mechanisation of the slaughter process, but to eliminate pain and suffering associated with the killing method, animals should always be unconscious at the time of slaughter.

In reality however, slaughterhouse designs are often suboptimal, stunning equipment is used at insufficient voltages, or is poorly maintained, resulting in ineffective stunning, and high line speeds and time constraints continue to place slaughterhouse staff under considerable pressure. Within such systems, animal stress, fear, pain and suffering is inevitable.

Various undercover investigations have revealed inhumane treatment, and even blatant abuse, of animals, in slaughterhouses. Since 2009, British animal advocacy organization Animal Aid has covertly filmed from within ten randomly chosen British slaughterhouses. They found evidence of cruelty and law breaking in nine of them. Their footage reveals animals being kicked, slapped, stamped on, picked up by fleeces and ears, and thrown into stunning pens. Animals are improperly stunned and killed, including by throat-cutting, while still conscious. Some are deliberately beaten, and pigs were filmed being burned with cigarettes [80]. 

The effects for both humans and animals caught up within these systems are deeply disturbing. Significant empirical evidence indicates that ongoing involvement in the commission of violent acts—particularly when required for economic or literal survival (as in the case of warfare [81,82])—can result in desensitisation toward violence in general, and increases in rates of the most violent crimes within surrounding communities. As noted by Kristina Mering in her study of Estonian slaughterhouse workers, “The core problem is the animal-industrial complex, the system of exploiting animals which also has negative effects on the workers in the system…” [33]. Today, such exploitative systems undoubtedly contribute to the disturbing rates of animal welfare abuses reported within modern slaughterhouses. In East London in the 1880s, they may well have contributed to the development of the world’s most infamous serial killer.

Stimulated by the disturbing slaughterhouse practices of the day, a broad urban-based animal welfare and slaughterhouse reform movement emerged in nineteenth-century Britain. Contemporary techniques based on the pole-axe, nape-stab, and Jewish ritual slaughter were too unreliable or too slow to ensure insensibility prior to exsanguination. And so stunning technologies such as captive bolt pistols were developed and tested throughout the nineteenth century. Humanitarian groups advocated the humane slaughter principle—that no animal should be slaughtered without first being stunned into insensibility. However, they were successfully opposed by the butchers’ trade organization, so the humane slaughter principle did not receive legislative sanction until the 1930s [83].

This old struggle between proponents and opponents of animal slaughtering reforms continues with similar vigour today. Within the UK, recent animal welfare abuses have led to the installation of CCTV, to monitor and improve animal welfare. According to the Food Standards Agency, 90% of British slaughterhouses now have CCTV installed. However, the footage is not monitored independently of the slaughterhouse business operator, and even the official veterinarians required to oversee the slaughtering and processing process are commonly refused access to it. As of January 2017, this deplorable situation persists, despite the serious objections of the British Veterinary Association (BVA) and the Veterinary Public Health Association (VPHA). BVA President Sean Wensley stated, “It is unacceptable that there are slaughterhouses that are not willing to share CCTV footage with official veterinarians. We are lobbying for CCTV to be mandatory in all slaughterhouses and for legislation to ensure that footage is readily available to vets” [84].

## 13. Conclusions

The brutality with which Jack the Ripper despatched and subsequently mutilated his victims in the 1880s was unprecedented within modern history, and plunged East London into a climate of fear. Widespread media reporting of Jack’s crimes, combined with the inability of the police to catch him, resulted in a fascination with the case that remains strong to this day.

Jack’s ability to rapidly locate and remove specific organs from several of his victims, in conditions of haste and very poor light, led to theories that he must have been surgically trained. However, re-examination of a mortuary sketch of one of his victims has revealed key aspects of the incisional technique used that are highly inconsistent with professional surgical training. Related discrepancies are also apparent in the language used within the only letter from Jack considered probably authentic.

Furthermore, the throat-cutting technique used to kill his victims, combined with Jack’s undoubted propensity for anatomical dissection with a knife, were highly consistent with the skillset of slaughterers of the times. And indeed, a very large number of small-scale slaughterhouses existed within the districts in which the murders occurred. The harsh socioeconomic conditions of the times may have influenced how the animals and their body parts were treated, as well as the subsequent behaviour of the murderer. We will never know for certain, but it is highly likely that Jack the Ripper honed the physical skills, and the psychological and behavioural attributes employed on his victims to such devastating effect, during his employment as a slaughterhouse worker.

These insights should stimulate a fundamental re-examination of the acceptability of animal slaughter today. Modern slaughterhouses are more humane in some ways than those of nineteenth-century East London; however, the vast, impersonal, mechanized killing operations of the twenty-first century are more desensitizing in others. With more than 70 billion terrestrial animals slaughtered annually in slaughterhouses of varying standards internationally by 2013 (The most recently reported year, by April 2017) [85], this represents one of the greatest animal welfare issues today.

Additionally, a considerable weight of recent sociological evidence indicates that those who commit violence towards animals are more likely to target people, and that rates of the most violent crimes are increased in communities surrounding slaughterhouses. Accordingly, the acceptability of animal slaughter should also be profoundly questioned on the basis of its potential human and societal impacts.

## References

[1] Evans S.P., Skinner K. (2000). The Ultimate Jack the Ripper Sourcebook: An Illustrated Encyclopedia.

[2] Bond T. (1888). Notes of Examination of Body of Woman Found Murdered & Mutilated in Dorset Street.

[3] Ryder S.P. Casebook: Jack the Ripper, 1996–2013. http://www.casebook.org.

[4] Marks K. (2006). Was Jack the Ripper a woman?. The Independent.

[5] Weston-Davies W. (2016). The Real Mary Kelly: Jack the Ripper’s Fifth Victim and the Identity of the Man that Killed Her.

[6] Sugden P. (2002). The Complete History of Jack the Ripper.

[7] Baranski A. (2009). Surgical Technique of Abdominal Organ Procurement.

[8] Vikram K., Maroju N.K. (2015). Exploratory Laparotomy. http://emedicine.medscape.com/article/1829835-overview.

[9] Carter H.V., Gray H. (1918). Diagram of sheath of Rectus above the arcuate line. Anatomy of the Human Body.

[10] Thibodeau G.A., Patton K.T. (2009). Linea alba. Mosby's Medical Dictionary.

[11] Roses R.E., Morris J.B., Zinner M.J., Ashley S.W. (2013). Incisions, closures and management of the abdominal wound. Maingot’s Abdominal Operation.

[12] Hamilton F.H. (1886). Principles and Practice of Surgery.

[13] Holme T. (1888). A Treatise on Surgery: Its Principles and Practice.

[14] Williams W. (1893). The Principles and Practice of Veterinary Surgery.

[15] Evans S.P., Skinner K. (2002). Jack the Ripper and the Whitechapel Murders.

[16] (1888). The London murders. Denbighshire Free Press.

[17] (1888). The murder of Annie J. Chapman. South Wales Echo.

[18] MacLachlan I. (2007). A bloody offal nuisance: The persistence of private slaughter-houses in nineteenth-century London. Urban History.

[19] (1888). The meat supply of London. Birmingham Daily Post.

[20] (1888). Horrible murder in Whitechapel. The Flintshire Observer.

[21] Odell R. (2006). Ripperology: A Study of the World’s First Serial Killer and a Literary Phenomenon.

[22] Barnett S.A. (1888). At last (letter). The Times.

[23] Camps F.E. (1966). More about “Jack the Ripper”. Lond. Hosp. Gaz..

[24] Shechita UK (2009). A Guide to Shechita.

[25] Velarde A., Rodriguez P., Dalmau A., Fuentes C., Llonch P., von Holleben K.V., Anil M.H., Lambooij J.B., Pleiter H., Yesildere T. (2014). Religious slaughter: Evaluation of current practices in selected countries. Meat Sci..

[26] Evans S.P., Rumbelow D. (2006). Jack the Ripper: Scotland Yard Investigates.

[27] Image in the Public Domain. https://en.wikipedia.org/wiki/From_Hell_letter#/media/File:FromHellLetter.jpg.

[28] Barnett S.A. (1889). Whitechapel horrors (letter). The Times.

[29] Wedderburn P. (2010). Dangerous dogs: Time for a new approach. Daily Telegraph.

[30] Seng P.M., Laporte R. (2005). Animal welfare: The role and perspectives of the meat and livestock sector. Rev. Sci. Tech..

[31] Jacques J.R. (2015). The slaughterhouse, social disorganization, and violent crime in rural communities. Soc. Anim..

[32] Cohidon C., Morisseau P., Derriennic F., Goldberg M., Imbernon E. (2009). Psychosocial factors at work and perceived health among agricultural meat industry workers in France. Int. Arch. Occup. Environ. Health.

[33] Leenaert T. (2016). Slaughterhouse Workers: The Meat Industry’s Other Victims. http://veganstrategist.org/2016/08/05/slaughterhouse-workers-the-meat-industrys-other-victims/.

[34] Fitzgerald A.J., Kalof L., Dietz T. (2009). Slaughterhouses and increased crime rates: An empirical analysis of the spillover from “The Jungle” into the surrounding community. Organ. Environ..

[35] U.S. Bureau of Labor Statistics (2005). Table SNR01: Highest Incidence Rates of Total Nonfatal Occupational Injury and Illness Cases, Private Industry, 2003.

[36] U.S. Bureau of Labor Statistics (2005). Table SNR06: Highest Incidence Rates of Total Nonfatal Occupational Injury Cases, Private Industry, 2003.

[37] Cortes K. (2005). Do Immigrants Benefit from An Increase in the Minimum Wage Rate? An Analysis by Immigrant Industry Concentration.

[38] U.S. Census Bureau, Economic Planning and Coordination Division (2006). County Business Patterns.

[39] Broadway M.J. (1990). Meatpacking and its social and economic consequences for Garden City, Kansas in the 1980s. Urban Anthropol..

[40] Broadway M.J. (2000). Planning for change in small towns or trying to avoid the slaughterhouse blues. J. Rural Stud..

[41] Stull D., Broadway M. (2004). Slaughterhouse Blues: The Meat and Poultry Industry in North America.

[42] Pinel P. (1962). Treatise on Insanity.

[43] Macdonald J.M. (1961). The Murderer and His Victim.

[44] Mead M. (1964). Cultural factors in the cause and prevention of pathological homicide. Bull. Menn. Clin..

[45] Wright J., Hensley C. (2003). From animal cruelty to serial murder: Applying the graduation hypothesis. Int. J. Offender Ther. Comp. Criminol..

[46] Ressler R., Burgess A., Douglas J. (1988). Sexual Homicides: Patterns and Motives.

[47] American Psychological Association (1987). Diagnostic and Statistical Manual of Mental Disorders.

[48] American Psychological Association (1994). Diagnostic and Statistical Manual of Mental Disorders.

[49] American Psychological Association (2000). Diagnostic and Statistical Manual of Mental Disorders.

[50] American Psychological Association (2013). Diagnostic and Statistical Manual of Mental Disorders.

[51] Gleyzer R., Felthous A.R., Holzer C.E. (2002). Animal cruelty and psychiatric disorders. J. Am. Acad. Psych. Law.

[52] Hensley C., Tallichet S.E., Dutkiewicz E.L. (2009). Recurrent childhood animal cruelty: Is there a relationship to adult recurrent interpersonal violence?. Crim. Justice Rev..

[53] Merz-Perez L., Heide K.M., Silverman I.J. (2001). Childhood cruelty and subsequent violence against humans. Int. J. Offender Ther. Comp. Criminol..

[54] Merz-Perez L., Heide K.M. (2004). Animal Cruelty: Pathway to Violence Against People.

[55] Ressler R.K., Burgess A.W., Hartman C.R., Douglas J.E., McCormack A. (1998). Murderers who rape and mutilate. J. Interpers. Violence.

[56] Tallichet S.E., Hensley C. (2004). Exploring the link between recurrent acts of childhood and adolescent animal cruelty and subsequent violent crime. Crim. Justice Rev..

[57] Verlinden S. (2000). Risk Factors in School Shootings. Ph.D. Thesis.

[58] Walters G.D. (2013). Testing the specificity postulate of the violence graduation hypothesis: Meta-analyses of the animal cruelty-offending relationship. Aggress. Violent Behav..

[59] Faver C.A., Strand E.B. (2003). To leave or to stay? Battered women’s concern for vulnerable pets. J. Interpers. Violence.

[60] Volant A.M., Johnson J.A., Gullone E., Coleman G.J. (2008). The relationship between domestic violence and animal abuse: An Australian study. J. Interpers. Violence.

[61] Carlisle-Frank P., Frank J.M., Nielsen L. (2004). Selective battering of the family pet. Anthrozoos.

[62] Ascione F.R., Weber C.V., Thompson T.M., Heath J., Maruyama M., Hayashi K. (2007). Battered pets and domestic violence: Animal abuse reported by women experiencing intimate violence and by nonabused women. Violence Against Women.

[63] Ascione F.R. (1998). Battered women’s reports of their partners’ and their children’s cruelty to animals. J. Emot. Abuse.

[64] Flynn C.P. (2000). Woman’s best friend: Pet abuse and the role of companion animals in the lives of battered women. Violence Against Women.

[65] Simmons C.A., Lehmann P. (2007). Exploring the link between pet abuse and controlling behaviors in violent relationships. J. Interpers. Violence.

[66] Flynn C.P. (2011). Examining the links between animal abuse and human violence. Crime Law Soc. Change.

[67] Arluke A., Levin J., Luke C., Ascione F.R. (1999). The relationship of animal abuse to violence and other forms of antisocial behavior. J. Interpers. Violence.

[68] Miller K.S., Knutson J.F. (1997). Reports of severe physical punishment and exposure to animal cruelty by inmates convicted of felonies and by university students. Child Abuse Negl..

[69] Felthous A.R., Kellert S.R. (1987). Childhood cruelty to animals and later aggression against people: A review. Am. J. Psychiatry.

[70] Cudworth E. (2015). Killing animals: Sociology, species relations and institutionalized violence. Sociol. Rev..

[71] Dillard J. (2008). A slaughterhouse nightmare: Psychological harm suffered by slaughterhouse employees and the possibility of redress through legal reform. Georget. J. Poverty Law Policy.

[72] Winders B., Nibert D. (2004). Consuming the surplus: Expanding “meat” consumption and animal oppression. Int. J. Sociol. Soc. Policy.

[73] Pachirat T. (2011). Every Twelve Seconds: Industrialized Slaughter and the Politics of Sight.

[74] Burt J. (2006). Conflicts around slaughter in modernity. Killing Animals.

[75] Eisenitz G. (1997). Slaughterhouse: The Shocking Tales of Greed, Neglect and Inhumane Treatment inside the U.S. Meat Industry.

[76] Shimshony A., Chaudry M.M. (2005). Slaughter of animals for human consumption. Rev. Sci. Tech..

[77] American Veterinary Medical Association (2016). AVMA Guidelines for the Humane Slaughter of Animals: 2016 Edition.

[78] World Organisation for Animal Health (OIE) (2016). Chapter 7.5. Slaughter of Animals. http://www.oie.int/international-standard-setting/terrestrial-code/access-online/.

[79] Farm Animal Welfare Council (2003). Report on the Welfare of Farmed Animals at Slaughter or Killing Part 1: Red Meat Animals.

[80] Animal Aid (2016). The “Humane Slaughter” Myth. http://www.animalaid.org.uk/h/n/CAMPAIGNS/slaughter/ALL///.

[81] Sanday P.R. (1981). The socio-cultural context of rape: A cross-cultural study. J. Soc. Issues.

[82] Archer D., Gartner R. (1987). Violence and Crime in Cross-National Perspective.

[83] MacLachlan I. (2005). Coup de grâce: Humane cattle slaughter in nineteenth-century Britain. Food Hist..

[84] Anon (2016). Vets Call for Unrestricted Access to Slaughterhouse CCTV. https://www.bva.co.uk/News-campaigns-and-policy/Newsroom/News-releases/Vets-call-unrestricted-access-slaughterhouse-CCTV/.

[85] Food and Agriculture of the United Nations FAOSTAT (Database): Livestock Primary (2017). http://www.fao.org/faostat/en/#data/QL.

